# KINAID: an orthology-based kinase-substrate prediction and analysis tool for phosphoproteomics

**DOI:** 10.1093/bioinformatics/btaf300

**Published:** 2025-05-10

**Authors:** Javed M Aman, Audrey W Zhu, Martin Wühr, Stanislav Y Shvartsman, Mona Singh

**Affiliations:** Computer Science Department, Princeton University, Princeton, NJ, 08544, United States; Lewis-Sigler Institute for Integrative Genomics, Princeton University, Princeton, NJ, 08544, United States; Lewis-Sigler Institute for Integrative Genomics, Princeton University, Princeton, NJ, 08544, United States; Department of Chemical and Biological Engineering, Princeton University, Princeton, NJ, 08544, United States; Lewis-Sigler Institute for Integrative Genomics, Princeton University, Princeton, NJ, 08544, United States; Department of Molecular Biology, Princeton University, Princeton, NJ, 08544, United States; Lewis-Sigler Institute for Integrative Genomics, Princeton University, Princeton, NJ, 08544, United States; Department of Molecular Biology, Princeton University, Princeton, NJ, 08544, United States; Flatiron Institute, New York, NY, 10010, United States; Computer Science Department, Princeton University, Princeton, NJ, 08544, United States; Lewis-Sigler Institute for Integrative Genomics, Princeton University, Princeton, NJ, 08544, United States

## Abstract

**Summary:**

Proteome-wide datasets of phosphorylated peptides, either measured in a condition of interest or in response to perturbations, are increasingly becoming available for model organisms across the evolutionary spectrum. We introduce KINAID (KINase Activity and Inference Dashboard), an interactive and extensible tool written in Dash/Plotly, that predicts kinase-substrate interactions, uncovers and displays kinases whose substrates are enriched amongst phosphorylated peptides, interactively illustrates kinase-substrate interactions, and clusters phosphopeptides targeted by similar kinases. KINAID is the first tool of its kind that can analyze data from not only *Homo sapiens* but also 10 additional model organisms (including *Mus musculus*, *Danio rerio*, *Drosophila melanogaster*, *Caenorhabditis elegans*, and *Saccharomyces cerevisiae*). We demonstrate KINAID’s utility by applying it to recently published *S. cerevisiae* phosphoproteomics data.

**Availability and implementation:**

Webserver is available at https://kinaid.princeton.edu; open-source python library is available at https://github.com/Singh-Lab/kinaid; archive is available at https://doi.org/10.24433/CO.8460107.v1.

## 1 Introduction

Protein phosphorylation and the ensuing signal cascading events are critical for many intracellular processes, including cell cycle, growth, and development. Altered signaling plays a role in various diseases, from cancer to immune disorders (Cohen 2001). Therefore, detailed knowledge of signaling is important to understand both normal cellular functioning as well as disease states. Mass spectrometry-based phosphoproteomics aims to map signaling networks by uncovering all phosphorylated peptides in a condition of interest and/or after a cellular perturbation (Savage and Zhang 2020). However, additional knowledge about substrate-kinase relationships is necessary to uncover the specific signaling events that led to the observed phosphorylated peptides. This is a challenging task; consequently, while tens of thousands of phosphosites have been identified across organisms (Hornbeck *et al.* 2019, Li *et al.* 2022), only 5% of known phosphosites have a kinase assigned to them (Needham *et al.* 2019).

**Figure 1.**
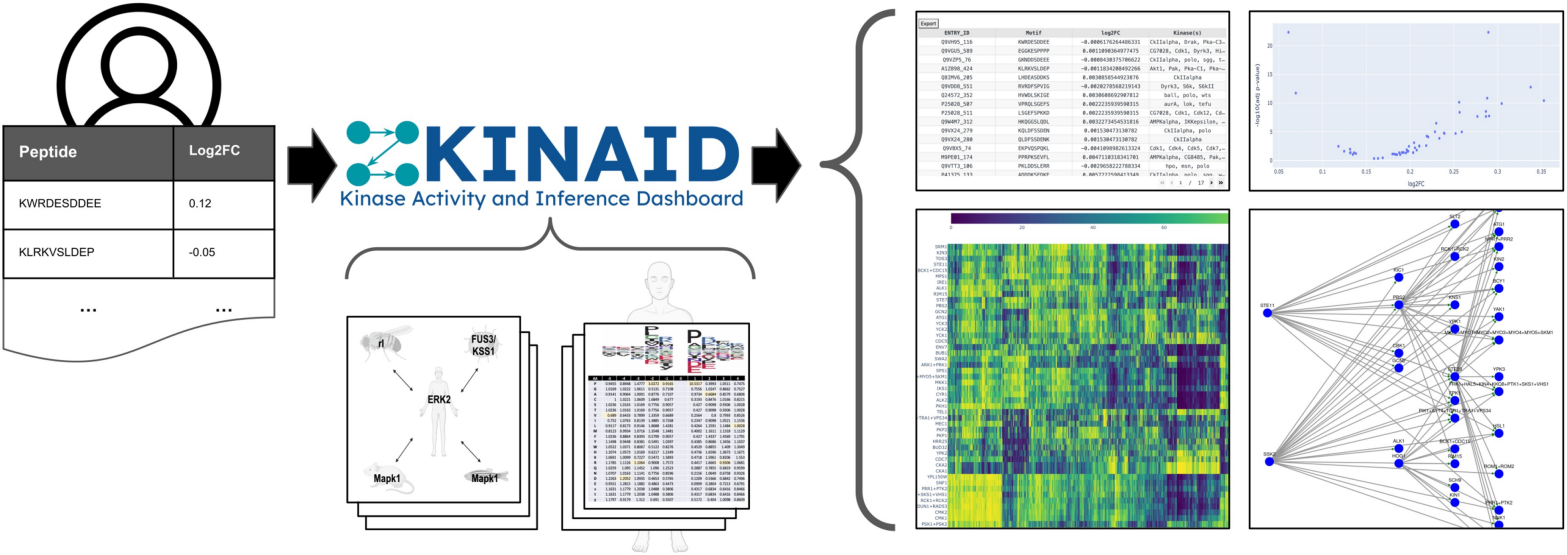
KINAID takes as input a file with phosphorylated peptide sequences, which can optionally have information about their log-fold changes in phosphorylation as compared to levels in a reference condition (top left), and processes it along with predetermined lists of kinase orthologs and position-weight matrices representing human kinase specificities (Johnson *et al.* 2023, Yaron-Barir *et al.* 2024) (center). The dashboard matches peptides to kinases and produces eight different tables and figures resulting from analyzing these data (right). Four examples are shown: a table of the kinases that match each peptide; a volcano plot of the average log-fold change in abundance of phosphorylated targets of each kinase versus the significance of this change; a heatmap depicting phosphopeptides clustered based on their matches to kinases; and a reconstructed phosphorylation network amongst kinases.

Here, we introduce the Kinase Analysis and Inference Dashboard (KINAID) to facilitate the study of phosphoproteomics experiments (Fig. 1). Given a set of phosphosite sequences (subsequences of ≥10 centered on a phosphosite), KINAID leverages experimentally determined specificities for human kinases and orthology information to uncover which kinases in the appropriate organism can phosphorylate these peptides. For phosphoproteomics experiments that include measurements for each peptide of the change in phosphorylation between conditions, KINAID determines the relative changes of kinase activities between these conditions. KINAID also provides interactive network visualizations of putative kinase-substrate interactions, along with clustering analysis of phosphosite sequences by the similarity of the kinases that can phosphorylate them. KINAID’s webserver supports *H. sapiens*, *M. musculus*, *D. rerio*, *D. melanogaster*, *C. elegans*, and *S. cerevisiae*, and the Python library additionally supports *R. norvegicus*, *X. tropicalis*, *A. gambiae*, *S. pombe*, and *A. thaliana*.

KINAID addresses a significant gap in phosphoproteomics analysis by offering a single platform with comprehensive capabilities to assign kinases to phosphosites across human and other model organisms, conduct kinase activity analyses, and create publication-ready figures in minutes. In contrast, many previous approaches for analyzing phosphoproteomics datasets either identify kinase-substrate interactions without further downstream analysis [review (Zhao *et al.* 2023)] or infer kinase activities while relying on known kinase-substrate relationships exclusively [review (Piersma *et al.* 2024)]. While some tools both identify kinase-substrate interactions and perform additional downstream analysis (e.g. Wiredja *et al.* 2017, Yilmaz *et al.* 2021, Johnson *et al.* 2024), they are focused on human or a single model organism. In contrast, KINAID supports 391 human, 384 mouse, 373 zebrafish, 268 worm, 178 fruit fly and 91 baker’s yeast kinases. Additionally, only PhosphoSitePlus (https://www.phosphosite.org/kinaseLibraryAction) leverages, as we do, the latest experimentally determined kinase specificities (Johnson *et al.* 2023, Yaron-Barir *et al.* 2024), which offer broader coverage and enhanced sensitivity to mutations within phosphosite sequences. KINAID is available both as a webserver and as a library that can be incorporated within users’ own software pipelines, whereas most tools (Yilmaz *et al.* 2021, Horn *et al.* 2014, Johnson *et al.* 2024) are only available via webservers. See Supplementary Table S1 for a comparison of KINAID to earlier software tools. KINAID is designed to be an easy-to-use and fast tool that can be readily incorporated as part of any phosphoproteomic pipeline.

## 2 Materials and methods

### 2.1 Input

KINAID requires as input (in either .tsv, .csv, or .xlsx) a set of phosphosite sequences (of length ≥10) with either the central residue being a phosphorylated S, T or Y or or an asterisk denoting the phosphorylated residue. The input can also optionally include a quantitative value for each peptide reflecting its change in phosphorylation in response to some perturbation (we assume that this is a ${\;\text{log}\;}_{2}$ fold-change in phosphorylation abundance, log2FC) as well as a *P*-value corresponding to the significance of this change. Additionally, the input sequences can be labeled by an ID (e.g. Uniprot, Entrez, Flybase, SGD, etc.), and by the position of the phosphorylated residue within the protein sequence with that ID. The user specifies the organism via a toggle menu.

### 2.2 Inferring kinase specificities via orthology mapping

The specificities for 303 Serine/Threonine and 93 Tyrosine human kinases are obtained from Johnson *et al.* (2023) and Yaron-Barir *et al.* (2024). For kinases in non-human organisms, we infer their specificities by determining their human orthologs and transferring the known specificities; in general, kinase specificity is strongly conserved across orthologs (Bradley *et al.* 2021), though specificities for duplicated kinases may diverge. We determine human orthologs using the integrative ortholog prediction tool DIOPT (Hu *et al.* 2011), which aggregates ortholog predictions made by numerous methods. In the case where multiple kinases in a model organism match the same human kinase, we combine all these kinases into a single family, as we cannot disambiguate their inferred specificities and targets. KINAID offers the users two modes: (i) one-to-one, where a single model organism kinase is matched to a single human kinase and (ii) ambiguous, which additionally includes kinases that have more complex orthology relationships to human kinases. Users wishing more conservative though lower coverage predictions of kinase-substrate interactions should choose the one-to-one option. A detailed description of the ortholog finding procedure is given in the Supplementary Methods. The number of kinases in each organism for which we have inferred specificities in both the one-to-one and ambiguous settings is shown in Supplementary Table S2. The median percent identities when comparing the model organism kinase domains with the kinase domains in their human orthologs are given in Supplementary Table S3. Percent identities are typically higher for pairs of one-to-one orthologs, as expected (Bradley *et al.* 2021). We note that while the KINAID web server is restricted to humans and five model organisms, the accompanying Python library allows users to perform the same analyses on DIOPT v9 supported organisms (Hu *et al.* 2011) with Serine/Threonine or Tyrosine kinases.

### 2.3 Matching peptides to kinases

For each phosphosite sequence, KINAID finds the kinases whose specificities have good matches to it using the position-specific scoring matrices (PSSM) provided by Johnson *et al.* (2023) and Yaron-Barir *et al.* (2024); these matches are found at runtime, thereby handling the case where phosphosite sequences contain variations from the reference proteome. For the phosphosite, its score is calculated separately using the “S/T favorability equation” and multiplied with the score of the rest of the sequence (Johnson *et al.* 2023); in short, “favorability” is the proportion of Serine to Threonine (or vice-versa) in the 10-mer defining the sequence. While KINAID allows the user to ignore the contributions of the phosphosite, by default we consider it, as we believe it is better to utilize the signal from this column in the PSSM.

For each kinase, we determine its background distribution of scores by using its PSSM to score all ∼89K phosphorylation sites in the Atlas of Human Kinase Regulation (Ochoa *et al.* 2016) for Serine/Threonine kinases or ∼7.3K sites in Yaron-Barir *et al.* (2024) for Tyrosine kinases. Then, once a phosphosite sequence of interest is scored using the PSSM of a particular kinase, we compare this score to the background distribution of scores for that kinase. As recommended in Johnson *et al.* (2023) and Yaron-Barir *et al.* (2024), queried sequences with a score within the top 10 percentile of the background distribution are considered to be targets of that kinase; the user can adjust the threshold to call a match. KINAID generates a single tabular file for all queried peptides and their matches. Phosphosite sequences that do not exceed the threshold for any kinase are ignored in all further downstream analyses.

### 2.4 Generated figures and tables

As KINAID uses the Plotly/Dash (https://plot.ly) library, the figures it creates can be easily magnified, cropped, and downloaded. All calculations are done server-side, allowing the application to handle large datasets. Moreover, all plots are generated on demand, meaning that the user “opens” the plot they are interested in and KINAID populates it, dramatically reducing the initial computation time. A tooltip describing each plot is provided when hovering over the section in the application. The following are brief descriptions of tables and figures generated by KINAID once the data is processed and the kinases are matched to the phosphosite sequences in the experiment.

*Match table:* A downloadable three-column table mapping sequences to a list of kinases predicted to phosphorylate it (Supplementary Fig. S1).

*Match count bar plot:* Bar plots depicting the number of matches for each kinase, sorted by magnitude (Supplementary Fig. S2).

*Phospshosite sequence log-fold change volcano plot for selected kinases:* Scatter plot comparing the provided log2FC phosphorylation of the phosphosite sequences (*x*-axis) against their corresponding *P*-values (*y*-axis). KINAID allows the user to specify subsets of kinases, and changes the color of peptides that are targets of these kinases, thus visualizing the trend of targets of specific kinases as having increased or decreased phosphorylation (Supplementary Fig. S3).

*Heatmap of phosphosite sequence based on kinase matches:* Heatmap depicting phosphosite sequences and the kinases that match them. Kinases are given on the *y*-axis and peptides on the *x*-axis. The cells are colored by the matching percentile scores between the substrate and kinase relative to the background distributions. The rows and columns are hierarchically clustered using Ward’s method (Ward 1963) with Euclidean distance (Supplementary Fig. S4).

*Kinase activity barplots:* Barplots depicting the relative activities of kinases (Supplementary Fig. S5). The activity of each kinase is computed using the *z*-test, which compares the mean log2FC of phosphorylation of its targets as compared to the mean log2FC of phosphorylation for all peptides in the experiment [see Casado *et al.* (2013) and Supplementary Methods]. For each kinase activity value, a one-sided *P*-value is computed, and multiple hypothesis test correction is performed using the Benjamini-Hochberg procedure (Benjamini and Hochberg 1995). Users can specify an FDR threshold (default 0.10) to highlight significant kinases.

*Kinase activity volcano plots:* A 2D version of the kinase activity barplot, where the *x*-axis is the average log2FC in phosphorylation of the targets of each kinase and the *y*-axis is the negative log of the adjusted *P*-value from the *z*-test calculation (Supplementary Fig. S6).

*Interactive kinome network reconstruction:* Assuming the user provides the IDs of the sequence in a supported format, KINAID generates a graphic of a network with nodes as kinases and edges existing where a phosphorylation event is predicted (Supplementary Fig. S7). The user has three additional options: choosing the subset of kinases to include in the network, the matching threshold, and a toggle to display the non-kinase substrates. Moreover, kinases not in the user-provided list are added to the network if they phosphorylate any of the kinases in the list.

*Full kinase network:* Visualization of the full network consisting of all predicted interactions between kinases. Increasing the matching threshold restricts matches to a higher percentile, thus reducing the number of edges in the network.

## 3 Results

*Runtime.* We apply KINAID to published human, mouse, zebrafish, fly, worm and yeast datasets to assess its scalability, versatility, and performance. All tests were conducted on a MacBook Pro (2.3 Ghz Intel i9, 32 GB of RAM) using the offline library of KINAID. Processing times are shown in Supplementary Table S4. The yeast test dataset of 5105 phosphosite sequences (Leutert *et al.* 2023) takes 11 s to score and match peptides, and the most extensive test dataset of nearly 30K mouse phosphosite sequences (Huttlin *et al.* 2010) takes 1.5 min.

*Case study.* We analyzed three experiments in yeast from Leutert *et al.* (2023) that are expected to perturb the well-studied HOG1, SNF1, TOR2 kinase pathways. Reassuringly, KINAID uncovers that the predicted targets for these kinases have significantly up-regulated phosphorylation (Supplementary Table S5). We investigate the HOG1 pathway further by generating a kinase-kinase network (Supplementary Fig. S7); we find excellent concordance with the curated network from Mosbacher *et al.* (2023). The plots generated for the HOG1 experiment are shown in Supplementary Section 3.

## 4 Conclusion

KINAID is a fast, flexible, and comprehensive tool for the analysis of high-throughput phosphoproteomics data. To date, it is the only kinase analysis tool that supports a large variety of organisms, performs kinase-substrate matching at the phosphosite sequence level, and provides enrichment analysis. The dashboard achieves fast performance in assigning substrates to their kinases and generates publication-ready figures. KINAID is open-source and can be updated with minimal effort for bespoke plots using the extensive Plotly library. In the future, as specificities for additional kinases are determined, KINAID can be easily extended. Moreover, as kinase-substrate prediction approaches become more sophisticated and accurate, KINAID can easily be adapted to utilize them. In conclusion, KINAID is a scalable, extensible framework that will be a great aid for analyzing phosphoproteomics experiments.

## Supplementary Material

btaf300_Supplementary_Data

## Data Availability

A reproducible CodeOcean capsule with all the accompanying data of this study is provided here at https://doi.org/10.24433/CO.8460107.v1
