# Supplementary material for: KINAID: an orthology-based kinase-substrate prediction and analysis tool for phosphoproteomics
Source: Bioinformatics. 2025 May 10;41(5):btaf300. doi: 10.1093/bioinformatics/btaf300 (PMC12122078; doi:10.1093/bioinformatics/btaf300)
Supplement: btaf300_Supplementary_Data [file btaf300_supplementary_data.pdf]

KINAID: an orthology-based kinase-substrate prediction and analysis tool for high-throughput phosphoproteomics

Javed M Aman
Audrey W Zhu
Martin Wühr
Stanislav Y Shvartsman
Mona Singh

1 Supplement

1.1 Supplementary Methods

1.1.1 Ortholog determination

We use DIOPT [Hu et al., 2011], a method that aggregates the results of up to 23 ortholog identification tools, to identify the best ortholog match for each of the kinases provided by Johnson et al. [2023] and Yaron-Barir et al. [2024]. We use API calls to DIOPT (`https://www.flyrnai.org/tools/diopt/web/diopt_api/v9/get_orthologs_from_entrez/none`); since different ortholog detection methods may not always agree, we process DIOPT's hits as described below.

For each human kinase with known specificity, we use DIOPT to search for its orthologs in a given species A. For each such query, DIOPT gives the candidate orthologous proteins (hits) in A along with five scores for each candidate: raw score, weighted score, best match, best match reverse, and confidence. The *raw score* is the number of methods that agree on the match in the given direction(s). The *weighted score* is similar to the *raw score* but DIOPT weighs each method based on overall functional similarities of orthologs [Hu et al., 2011]. DIOPT gives directionality for each hit; these are indicated by the binary variables *best match* (i.e., the sequence in A is a best match for the human sequence) and *best match reverse* (the human sequence is the best for the given sequence in A). *Confidence* can be three values: *high*, where both both best match and best match reverse are true; *medium* where exactly one of best match or best match reverse is true, or alternatively raw score ≥ 2; or *low*, in which case KINAID does not consider the hit further.

For each human kinase, KINAID chooses the orthologs from the list of hits in species A returned by DIOPT, by ranking the hits hierarchically with the aforementioned scores. First, for all hits in species A, we use DIOPT to search for its orthologs in human; we do this in order to remove those with a high confidence match to a human kinase for which we do not know specificity. Then, for each human kinase, we rank the hits by confidence, then by best match reverse, then by best match, and finally by weighted score. The ortholog(s) in species A with the top rank is chosen as the match for the human kinase. By default, KINAID restricts to one-to-one mapping between species A and human kinases. The user can turn the feature off, allowing more complex orthology relationships. If multiple hits in A match to the same human kinase (i.e., hits have the same rank), KINAID aggregates the set of kinases in A and denotes it in figures with an identifier consisting of "$\sim$", the name of the first symbol (alphabetically) in the set, and the suffix "-like". If multiple human kinases match the same ortholog in A, KINAID will choose a single human kinase. In particular, KINAID will now perform the same ranking procedure across these multiple human proteins (instead of just within a single one), and the ortholog in A with the highest rank is chosen; however, in the situation when there is a tie in weighted scores, the *top of list* (by DIOPT) ortholog is chosen. The numbers of kinases in fly, mouse, worm, yeast, and zebrafish that have this type of tie are 11, 0, 33, 10, and 0, respectively.

1.1.2 Kinase activity z-score

To uncover the activity of each kinase, we use the z-test which compares the mean log2FC of phosphorylation of its targets as compared to the mean log2FC of phosphorylation for all phosphosite sequences in the experiment [Casado et al., 2013, Hernandez-Armenta et al., 2017]. For each kinase k, we compute the one-sided z-test statistic, z_k as $\frac{x_k-\mu_0}{\sigma_0/\sqrt{n_k}}$, where x_k is the average log2FC of the targets that match kinase k, n_k is number of targets that match kinase k, and μ_0 and σ_0 are the average log2FC and standard deviation, respectively, of all detected phosphosite sequences. We compute the p-value of over-enrichment (i.e., $z_k > 0$) as 1-norm.cdf(z_k), and under-enrichment as norm.cdf(z_k).

2 Supplementary Tables

**Name**	**Lib**	**Substrate ID Method**	**Kin. Act.**	**Net. Viz.**	**Organisms**
PhosphoSitePlus [Johnson et al., 2024]	N	Position-specific scoring matrices	Y	N	Human
RoKAI† [Yilmaz et al., 2021] [Yilmaz et al., 2023]	N	Functional network	Y	Y	Human, Rat
KSEA [Wiredja et al., 2017]	Y	Known substrates & NetworkKin [Linding et al., 2008]	Y	N	Human
KinomeXplorer [Horn et al., 2014]	N	NetPhorest [Miller et al., 2008] & NetworkKin [Linding et al., 2008]	N	N	Human, Yeast
KINAID	Y	Position-specific scoring matrices	Y	Y	Human, Yeast, Fly, Worm, Mouse, Zebrafish

Table 1: Related phosphoproteomic analysis software tools. The columns give, respectively, the name of the software application; if it provides a software library along with a website; a description of how it identifies substrates of kinases; if it uncovers which kinases are active in a condition; whether it can produce kinase-substrate visualizations; and what organisms it supports. †RoKAI includes RoKAIXplorer [Yilmaz et al., 2023].

	# kinases with inferred specificities				# human kinase matrices utilized	
	1-to-1		ambiguous			
**organism**	S/T	Y	S/T	Y	S/T	Y
fly	118	30	144	38	137	37
mouse	294	91	296	91	295	91
worm	112	25	234	39	151	35
yeast	56	2	90	4	78	4
zebrafish	272	86	288	88	280	87
clawed_frog†	267	83	278	87	271	85
fission_yeast†	55	2	84	5	75	5
mosquito†	105	22	133	36	132	35
rat†	293	91	295	91	294	91
thale_cress†	40	6	255	81	107	28

Table 2: For each model organism, we give the number of Serine/Threonine (S/T) and Tyrosine (Y) kinase sequences for which KINAID has inferred specificities. If a kinase is dual specificity, it is counted in both the S/T and Y columns. We give these numbers both for when KINAID is used in 1-to-1 mode (where there is a single sequence in the model organism that matches a single human sequence) and in ambiguous mode (where there are either 1-to-1 relationships or when multiple kinases in the model organism match the same human sequence). The number of S/T and Y kinase specificity matrices utilized gives the distinct number of human matrices used to infer for the specificities for the Serine/Threonine and Tyrosine kinases in the model organism when KINAID is used in ambiguous mode. When KINAID is used in 1-to-1 mode, the number of human kinases utilized is the number of kinases in the model organism with inferred specificities. The approximate number of known kinases for fly, mouse, worm, yeast, and zebrafish are 221 [Morrison et al., 2000], 540 [Caenepeel et al., 2004], 428 [Girard et al., 2007], 129 [Breitkreutz et al., 2010], and 692 [Wlodarchak et al., 2016], respectively. We sum the total number S/T and Y for each organism and calculate coverage for both modes. The respective number of 1-to-1 coverage is 67%, 71%, 32%, 45%, and 51%. In the ambiguous mode the coverages grow to 87%, 72%, 65%, 73%, 55%, respectively. †These species are available only in the KINAID Python library.

	Median Identity w/ Human (S/T)				Median Identity w/ Human (Y)	
	1-to-1		ambiguous		1-to-1	ambiguous
**organism**	SDR	domain	SDR	domain	domain	domain
rat†	1.0	0.99	1.0	0.99	0.98	0.98
mouse	1.0	0.99	1.0	0.99	0.98	0.98
clawed_frog†	1.0	0.92	1.0	0.92	0.89	0.89
zebrafish	1.0	0.90	1.0	0.90	0.85	0.85
fly	0.93	0.69	0.93	0.68	0.62	0.6
mosquito†	0.93	0.7	0.93	0.69	0.62	0.61
worm	0.87	0.62	0.6	0.44	0.5	0.46
fission_yeast†	0.71	0.5	0.67	0.49	0.32	0.33
yeast	0.67	0.48	0.64	0.45	0.33	0.33
thale_cress†	0.67	0.5	0.57	0.41	0.33	0.29

Table 3: For each organism, we give the median sequence identity of kinases with their human orthologs. The kinases are split into S/T and Y kinases, as well as those that have 1-to-1 or more complicated orthology relationships with their human counterparts. Percent identity is determined by comparing the match states of the kinase domains (PF00069_Pkinase) from the orthologous pair. For the S/T kinases, we add a column giving the percent identity in 15 structurally conserved specificity-determining residues (SDR), as determined in Bradley et al. [2021]; that is, the substrate specificity of S/T kinases is thought to be determined by these residues. The organisms are sorted roughly by their evolutionary distances to human. †These species are available only in the KINAID Python library.

Organism	Publication	# Phosphosites	Time to score & match (sec)
fly	Yang et al. [2024]	4139	7.98
human	Humphrey et al. [2013]	5936	17.48
mouse	Huttlin et al. [2010]	29978	92.41
worm	Li et al. [2021]	4061	6.28
yeast	Leutert et al. [2023]	5258	6.01
zebrafish	Kwon et al. [2015]	1025	3.32

Table 4: Processing times of KINAID on sample published datasets [Humphrey et al., 2013, Huttlin et al., 2010, Li et al., 2021, Leutert et al., 2023, Kwon et al., 2015] for each of the model organisms. Data from each experiment is processed on a 2019 MacBook Pro 2.3Ghz with 32GB of RAM using the KINAID library. Note that the specificity matrices, backgrounds, and orthologs were loaded once for all experiments (approximately 20 seconds). The provided processing time is the total for scoring the phosphosite sequences using the matrices and matching the sequences by ranking their scores as compared to background sequences in the Ochoa et al. [2016] and Yaron-Barir et al. [2024] backgrounds. When a phosphosite sequence is not provided (or is incomplete), the sites are extracted from the organism's proteome from UniProt.

Upstream kinase	Experiment label	Mean log2FC of substrates	Adj p-value
HOG1	KC	0.29	1.5e-04
SNF1	GL	0.20	1.1e-2
TOR2	DP	0.05	3.1e-2

Table 5: KINAID evaluated on yeast phosphoproteomic experiments from Vieitez et al. [2022] Three different experiments, potassium-chloride (KC), glycerol (GL), and diploid strain (DP), were expected to activate the HOG1, SNF1, and TOR2 pathways, respectively. In all three cases, there is a significant (Benjamini-Hochberg [Benjamini and Hochberg, 1995] adjusted p-values) increased phosphorylation of the predicted targets of those kinases, as computed via the z-test.

3 KINAID's figures when run on the HOG experiment from Leutert et al. (2023) with ambiguous mapping

Export

SGD:S000001915_1313	HSFGKYLEVMF	
SGD:S000003045_12	NASGGYMQPDQ	KIN3,PBS2,SWE1
SGD:S000003170_493	LGDTLYEEFGI	
SGD:S000003483_162	SVSNSYSASDE	
SGD:S000001136_19	NSSNSYELESG	PBS2
SGD:S000001397_276	IISRRYSNTTI	
SGD:S000001498_18	SKQPAYVNKQP	
SGD:S000001678_348	KYPEHYAEQLK	
SGD:S000003941_297	FKGKAYLAQSP	KIN3
SGD:S000004239_1037	ELGDAYVSSDE	PKP2,PBS2
SGD:S000004306_43	GVDSQYTNGTQ	
SGD:S000004327_15	IQRETYDSNES	PBS2
SGD:S000004784_346	SNNDSYGSNND	PBS2
SGD:S000004784_338	KKKSSYGSSNN	PKP2
SGD:S000004784_312	KKKSSYGSNND	PKP2
SGD:S000004842_185	VEDAEYESSDD	PKP2,PBS2
SGD:S000005021_528	HDINGY_____	SWE1

1 / 22

Figure 1: **Match table.** The first page of the match table generated when using the example dataset. The first column indicates the ID of the phosphosite which consists of the SGD ID of the yeast protein followed by an underscore and the position of the phosphosite. The second column is the sequence used by KINAID around the phosphosite. The third column gives the kinases that are predicted to match that sequence.

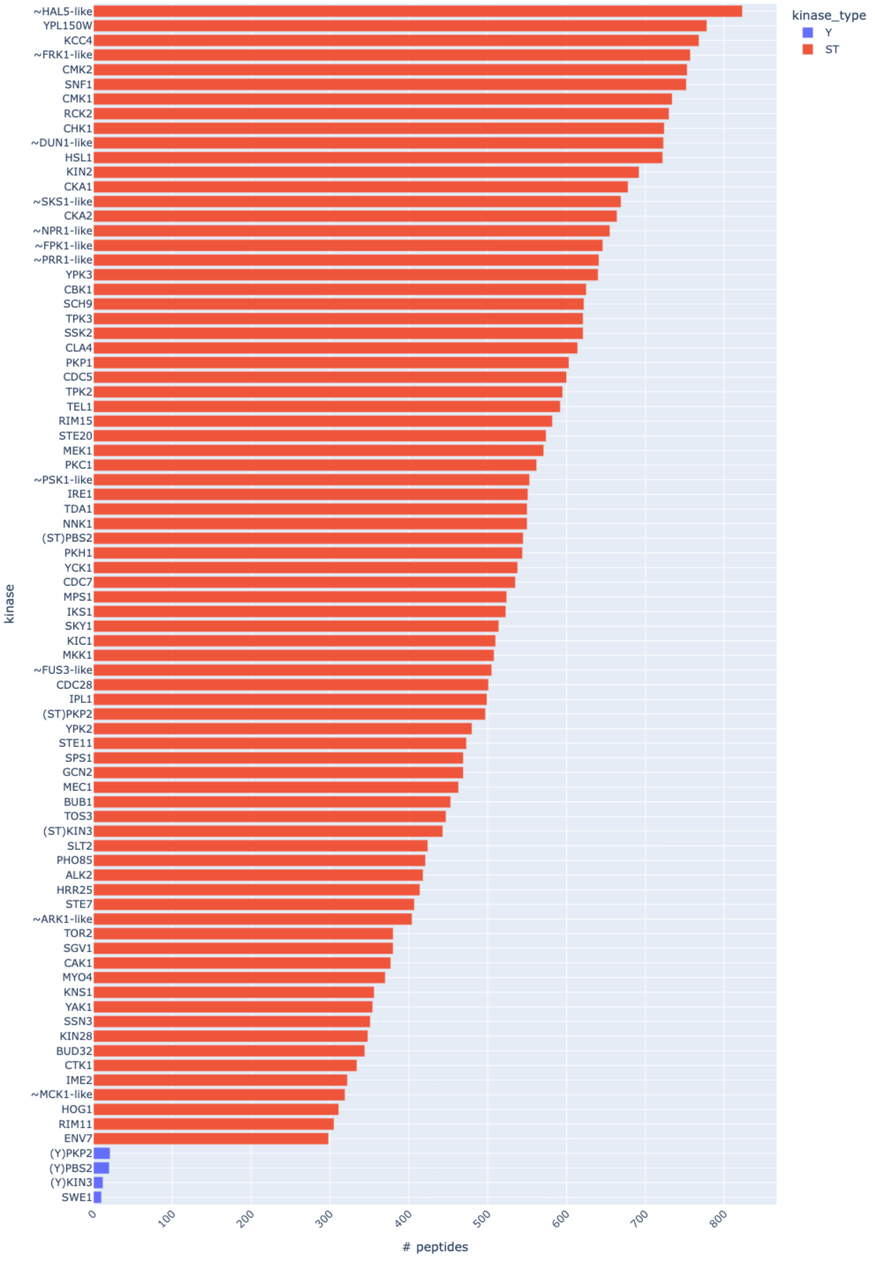

Figure 2: **Match count bar plot.** A bar plot of the number of phosphosites in the example experiment that match a given kinase. The kinases are sorted by the number of distinct phosphosites they match. Red indicates Serine/Threonine kinases while blue indicates Tyrosine kinases. In the case of dual-specificity kinases (e.g., PBS2) a prefix of either (ST) or (Y) represents the context.

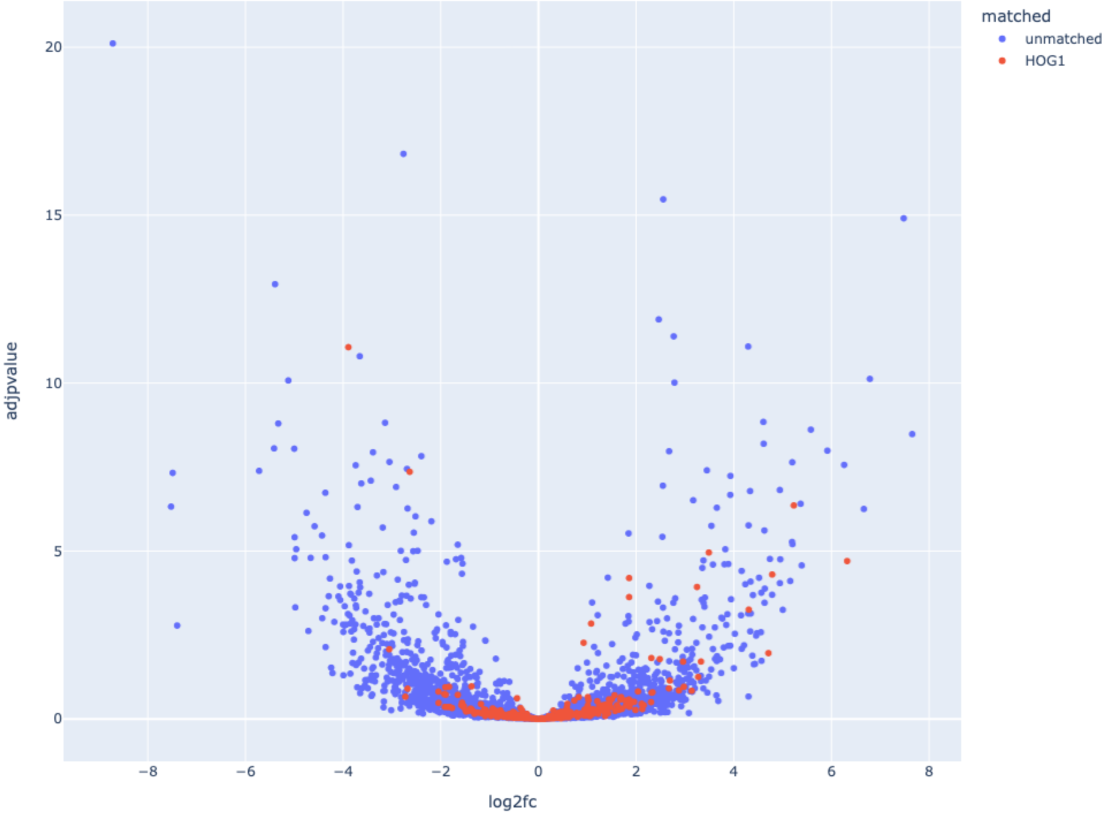

Figure 3: **Peptide log-fold change volcano plot for selected kinases.** A volcano plot where each dot corresponds to the phosphosite phosphorylation activity (x-axis) versus its adjusted p-value depicting its change in phosphorylation activity, both calculated from the mass spectrometry experiment (and given as input to KINAID). Highlighted in red are the phosphosites predicted by KINAID to be targets of HOG1, since this data arises from experiment expected to affect the HOG pathway. The user can choose which kinase(s)'s substrates should be highlighted.

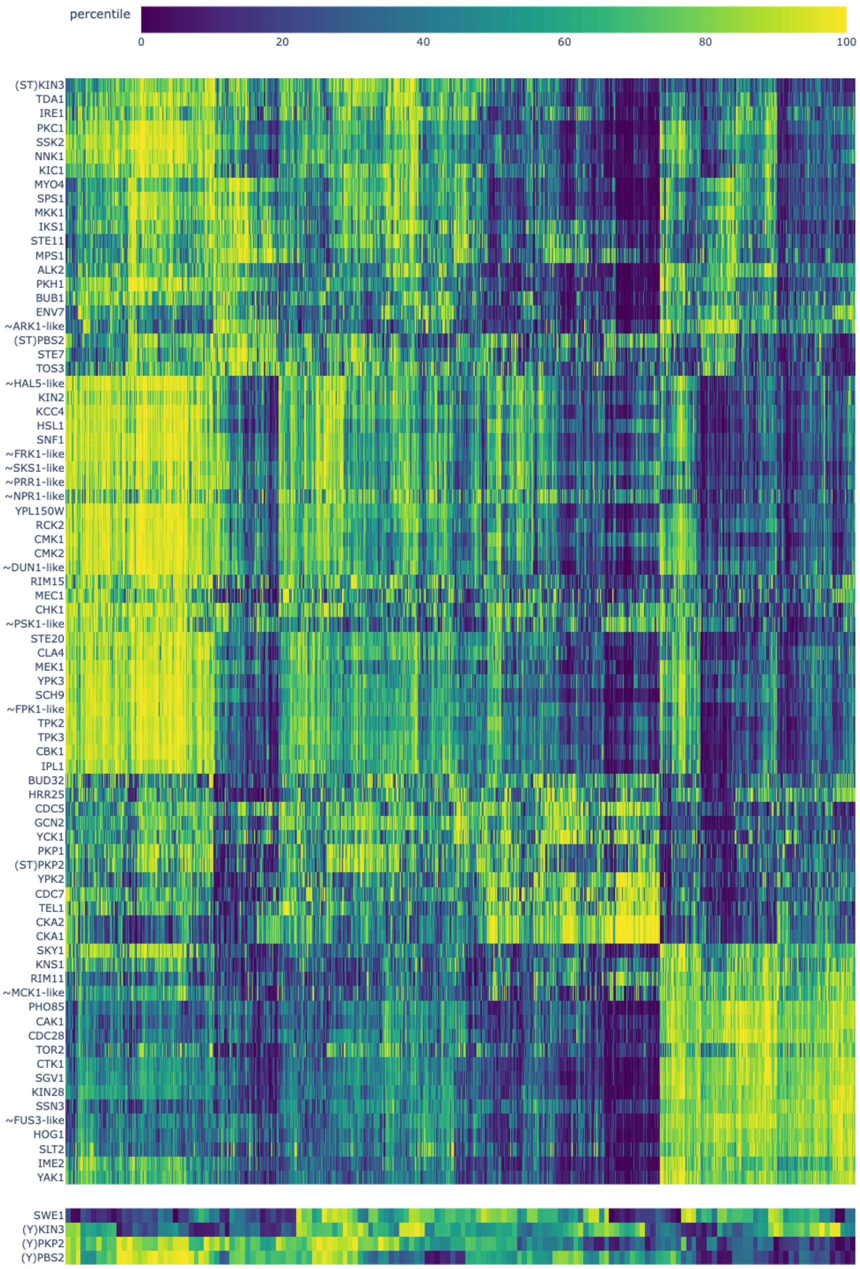

Figure 4: **Heatmap of phosphosite sequences based on kinase matches.** A heatmap of the percentile scores of phosphosites (columns) for each kinase (rows), computed as the percent of background sequence that score lower than this sequence when using the specificity matrix for the kinase. When hovering over a pixel, KINAID provides the ID of the phosphosite sequence as well as its score with the kinase of that row. Both rows and columns have been hierarchically clustered. There are two heatmaps, one for S/T kinases (top) and Y kinases (bottom). The (ST) or (Y) indicate the context of a dual-specificity kinases (e.g., PBS2).

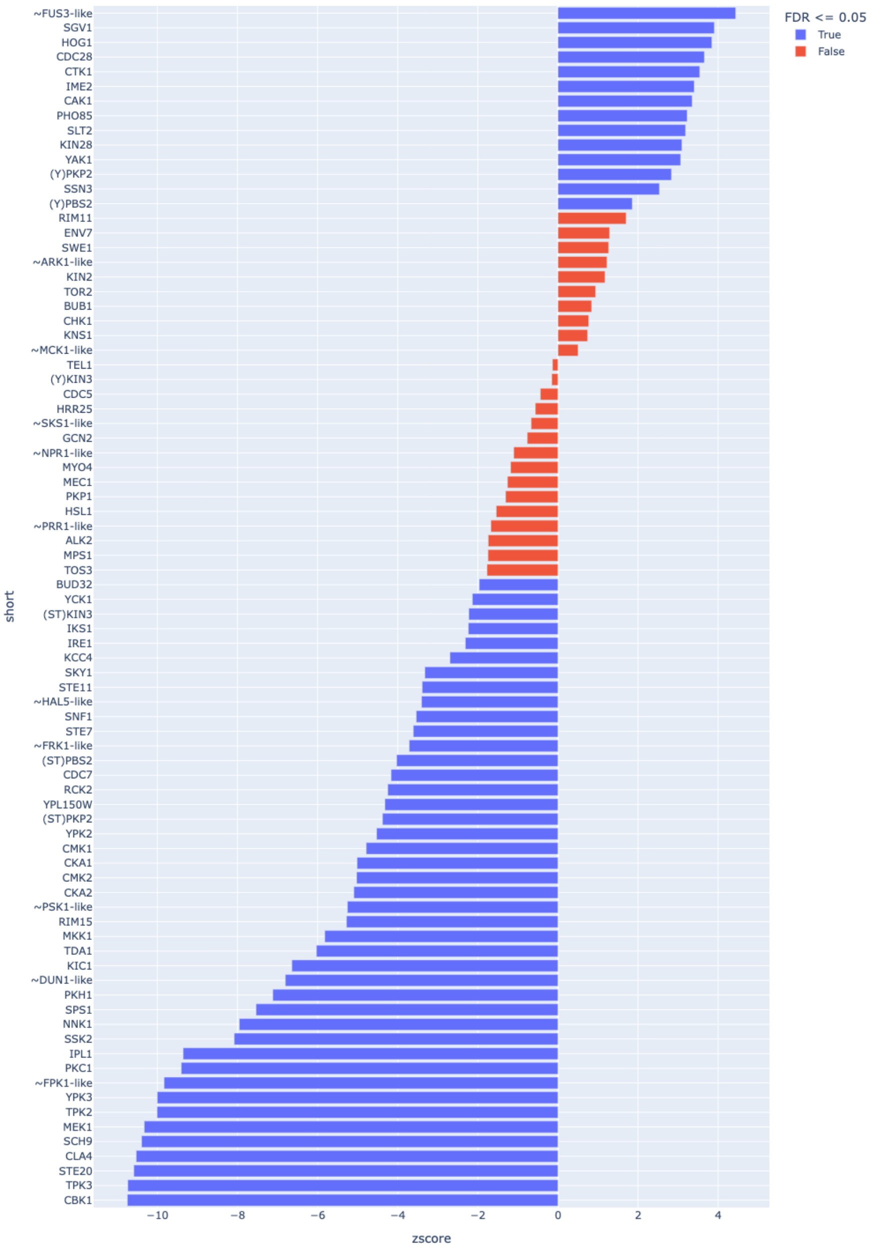

Figure 5: **Kinase activity barplots.** Kinases are sorted by their z-scores of activities (based on their predicted targets), and activities are depicted by a bar plot. The color of the bar indicates if the z-score is considered significant after Benjamini-Hochberg [Benjamini and Hochberg, 1995] FDR correction. Note that Figure 2 showed that HOG1 has a relatively low number of specific phosphosite matches; however, the activities of these peptides demonstrate that HOG1 is upregulated (third from top) compared to other kinases.

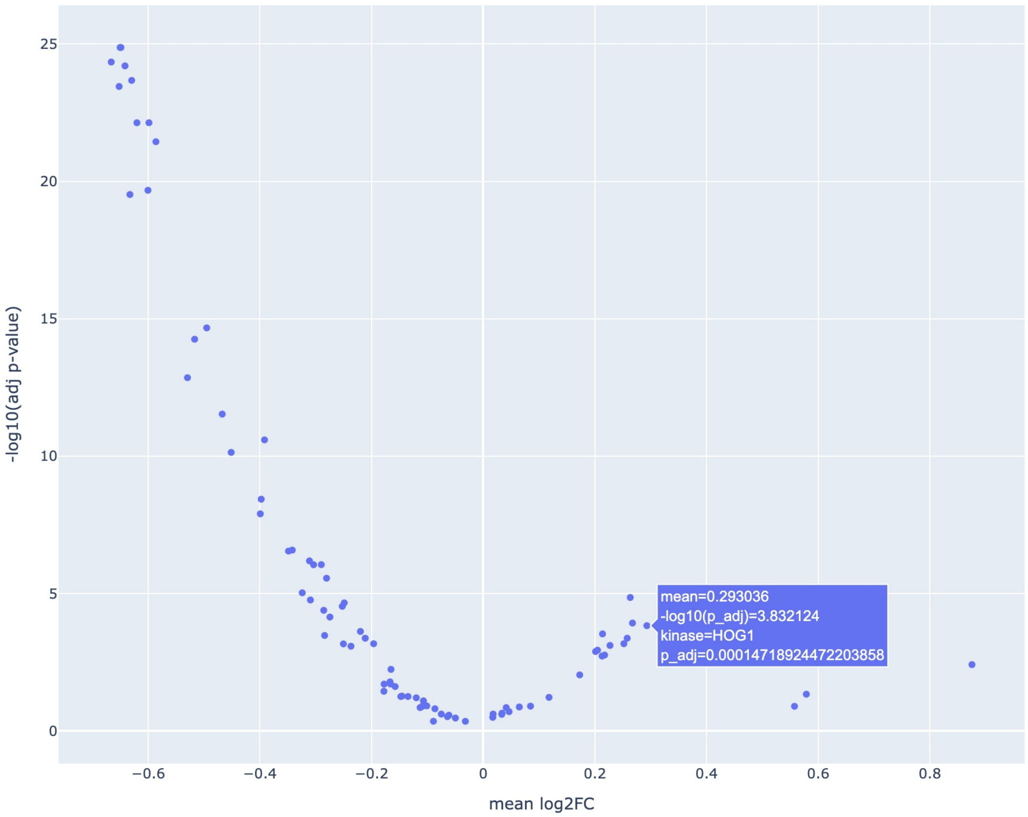

Figure 6: **Kinase activity volcano plots.** For each kinase, we show the mean log2FC of its targets (x-axis) and adjusted p-value of its enrichment as computed via the z-score. Hovering the mouse over a point shows statistics of the kinase represented. HOG1 is clearly seen as one of the more active kinases of S/T kinases, as it is towards the right. All kinases on the right of HOG1 are Tyrosine kinases.

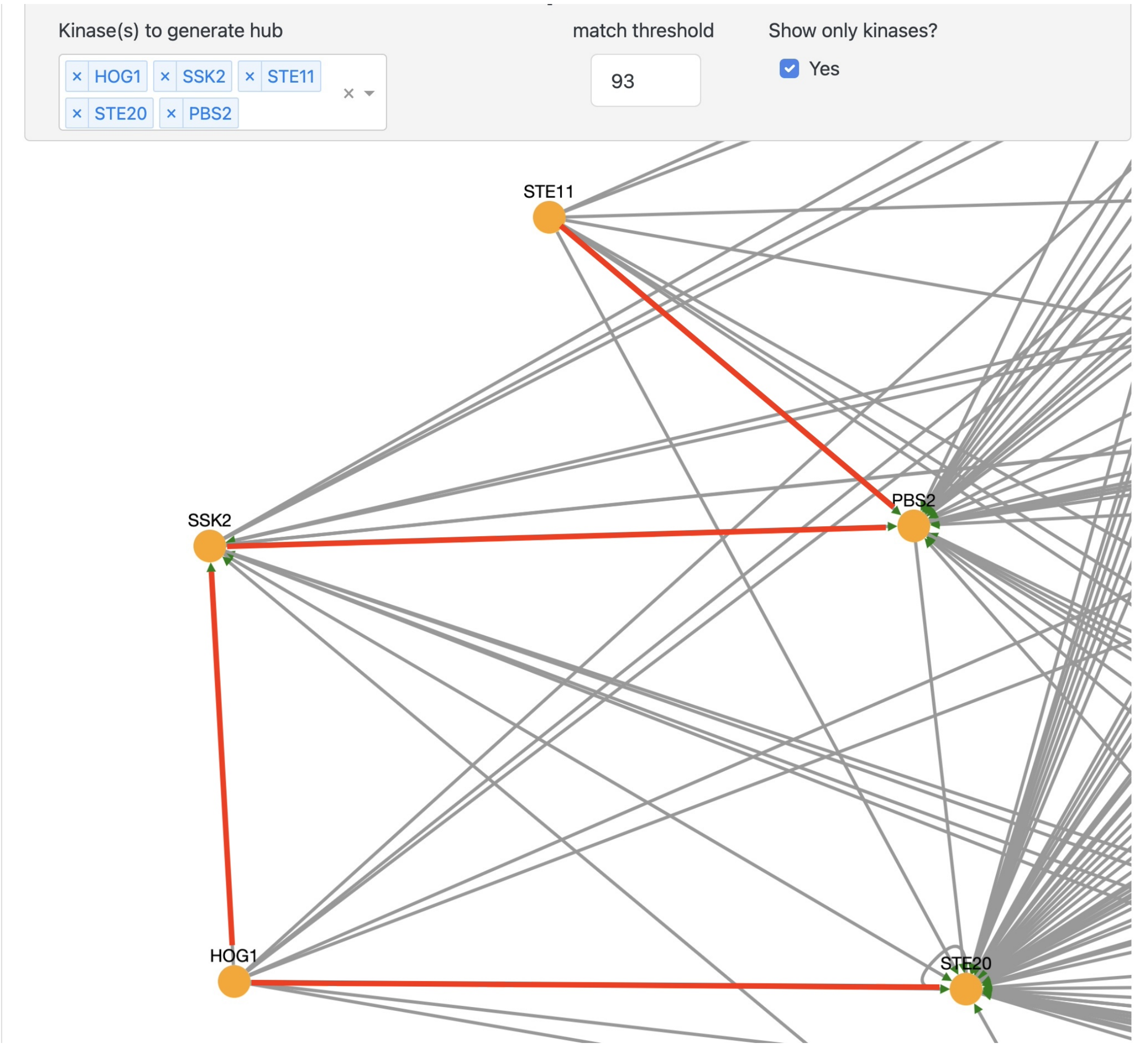

Figure 7: **Interactive kinome network reconstruction.** Network reconstruction by KINAID when providing kinases relevant for the HOG pathway, as specified by Mosbacher et al. [2023]: HOG1, STE20, STE11, PBS2, and SSK2. The kinase-substrate interactions involving these proteins that are mentioned by Mosbacher et al. [2023] are overlaid in red on top of KINAID's visualization in order to show that they are recapitulated via KINAID's analysis.

References

Yoav Benjamini and Yosef Hochberg. Controlling the false discovery rate: A practical and powerful approach to multiple testing. *Journal of the Royal Statistical Society: Series B (Methodological)*, 57(1):289–300, 1995.

David Bradley et al. Sequence and structure-based analysis of specificity determinants in eukaryotic protein kinases. *Cell Reports*, 34:108602, 01 2021.

Ashton Breitkreutz et al. A global protein kinase and phosphatase interaction network in yeast. *Science (New York, N.Y.)*, 328:1043–6, 05 2010. doi: 10.1126/science.1176495.

Sean Caenepeel et al. The mouse kinome: Discovery and comparative genomics of all mouse protein kinases. *Proceedings of the National Academy of Sciences of the United States of America*, 101: 11707–12, 09 2004. doi: 10.1073/pnas.0306880101.

Pedro Casado et al. Kinase-substrate enrichment analysis provides insights into the heterogeneity of signaling pathway activation in leukemia cells. *Science Signaling*, 6:rs6, 03 2013.

Lisa Girard et al. WormBook: The online review of Caenorhabditis elegans biology. *Nucleic acids research*, 35:D472–5, 02 2007. doi: 10.1093/nar/gkl894.

Claudia Hernandez-Armenta et al. Benchmarking substrate-based kinase activity inference using phosphoproteomic data. *Bioinformatics*, 33, 02 2017. doi: 10.1093/bioinformatics/btx082.

Heiko Horn et al. KinomeXplorer: An integrated platform for kinome biology studies. *Nature methods*, 11:603–4, 05 2014.

Claire (Yanhui) Hu et al. An integrative approach to ortholog prediction for disease-focused and other functional studies. *BMC Bioinformatics*, 12:357, 08 2011.

Sean Humphrey et al. Dynamic adipocyte phosphoproteome reveals that Akt directly regulates mtorc2. *Cell metabolism*, 17, 05 2013. doi: 10.1016/j.cmet.2013.04.010.

Edward L Huttlin et al. A tissue-specific atlas of mouse protein phosphorylation and expression. *Cell*, 143(7):1174–1189, 2010. ISSN 0092-8674.

Jared Johnson et al. An atlas of substrate specificities for the human serine/threonine kinome. *Nature*, 613:1–8, 01 2023.

Jared Johnson et al. PhoshoSitePlus kinase prediction tool, 2024. Accessed: 2024-10-30.

Oh Kwang Kwon et al. Global analysis of phosphoproteome dynamics in embryonic development of zebrafish (Danio rerio). *Proteomics*, 16, 10 2015. doi: 10.1002/pmic.201500017.

Mario Leutert et al. The regulatory landscape of the yeast phosphoproteome. *Nature Structural and Molecular Biology*, 30, 10 2023.

Wen-Jun Li et al. Insulin signaling regulates longevity through protein phosphorylation in Caenorhabditis elegans. *Nature Communications*, 12:4568, 07 2021. doi: 10.1038/s41467-021-24816-z.

Rune Linding et al. Networkin: A resource for exploring cellular phosphorylation networks. *Nucleic acids research*, 36:D695–9, 02 2008. doi: 10.1093/nar/gkm902.

M. Miller, L. Jensen, F. Diella, C. Jorgenson, M. Tinti, L. Li, et al. Linear motif atlas for phosphorylation-dependent signaling. *Science Signaling*, 1(35):ra2, 2008.

Deborah Morrison et al. Protein kinases and phosphatases in the Drosophila genome. *The Journal of cell biology*, 150:F57–62, 07 2000. doi: 10.1083/jcb.150.2.F57.

Maximilian Mosbacher et al. Positive feedback induces switch between distributive and processive phosphorylation of Hog1. *Nature Communications*, 14, 04 2023.

David Ochoa et al. An atlas of human kinase regulation. *Molecular Systems Biology*, 12:888, 12 2016.

Cristina Vieitez et al. High-throughput functional characterization of protein phosphorylation sites in yeast. *Nature Biotechnology*, 40:1–9, 03 2022. doi: 10.1038/s41587-021-01051-x.

Danica D Wiredja et al. The KSEA App: a web-based tool for kinase activity inference from quantitative phosphoproteomics. *Bioinformatics*, 33(21):3489–3491, 06 2017. ISSN 1367-4803.

Nathan Wlodarchak et al. Comparative analysis of the human and zebrafish kinomes: focus on the development of kinase inhibitors. *Trends in Cell and Molecular Biology*, 10:49–75, 03 2016.

Liu Yang, Audrey Zhu, et al. ERK synchronizes embryonic cleavages in Drosophila. *Developmental Cell*, 2024. ISSN 1534-5807. doi: https://doi.org/10.1016/j.devcel.2024.08.004.

Tomer Yaron-Barir et al. The intrinsic substrate specificity of the human tyrosine kinome. *Nature*, 629:1–8, 05 2024.

Serhan Yilmaz et al. Robust inference of kinase activity using functional networks. *Nature Communications*, 12, 02 2021.

Serhan Yilmaz et al. Making proteomics accessible: RokaiXplorer for interactive analysis of phosphoproteomic data. *bioRxiv*, 2023. doi: 10.1101/2023.08.22.553639. URL https://www.biorxiv.org/content/early/2023/08/23/2023.08.22.553639.
